# Personalizing Antidepressant Therapy: Integrating Pharmacogenomics, Therapeutic Drug Monitoring, and Digital Tools for Improved Depression Outcomes

**DOI:** 10.3390/jpm15120616

**Published:** 2025-12-10

**Authors:** Mikhail Parshenkov, Sergey Zyryanov, Galina Rodionova, Anna Dyakonova, Petr Shegay, Andrei Kaprin, Grigory Demyashkin

**Affiliations:** 1 Department of Digital Oncomorphology, National Medical Research Centre of Radiology, 2nd Botkinsky pass., 3, 125284 Moscow, Russia; m.parshenkov.research@gmail.com (M.P.);; 2 Laboratory of Histology and Immunohistochemistry, Institute of Translational Medicine and Biotechnology, FSAEI HE I.M. Sechenov First MSMU of MOH of Russia (Sechenovskiy University), Trubetskaya st., 8/2, 119048 Moscow, Russia; 3 Department of General and Clinical Pharmacology, Peoples’ Friendship University of Russia (RUDN University), Miklukho-Maklaya st., 6, 117198 Moscow, Russia; 4 Department of Pharmaceutical and Toxicological Chemistry Named After A.P. Arzamastsev, FSAEI HE I.M. Sechenov First MSMU of MOH of Russia (Sechenovskiy University), ave. Vernadsky, 96k1, 119526 Moscow, Russia; 5 Department of Urology and Operative Nephrology, Peoples’ Friendship University of Russia (RUDN University), Miklukho-Maklaya st., 6, 117198 Moscow, Russia

**Keywords:** major depressive disorder, pharmacogenetics, therapeutic drug monitoring, artificial intelligence, precision psychiatry, pharmacology

## Abstract

**Background**: Major Depressive Disorder (MDD) is a leading global health concern, yet its pharmacological management is hampered by a «trial-and-error» approach, with a significant proportion of patients failing to achieve remission with initial therapy. This challenge stems from the disorder’s marked biological heterogeneity, which is poorly captured by current broad diagnostic categories. This literature review synthesizes the latest evidence across three complementary fields poised to revolutionize MDD treatment: pharmacogenetics testing (PGT), therapeutic drug monitoring (TDM), and artificial intelligence (AI). We hypothesize that integrating all three facilitates the transition from empirical prescribing to model-informed precision dosing (MIPD), enabling prediction of optimal antidepressant selection and dosage before the first dose is administered. The convergence of these technologies, supported by an interdisciplinary framework, has the potential to enhance current treatment strategies and contribute to more individualized psychiatric care. **Conclusions**: Antidepressant therapy for MDD may be further optimized through the combined use of TDM, PGT, and digital tools. However, the development of this field requires ongoing research and interdisciplinary work.

## 1. Introduction

Major depressive disorder (MDD) is one of the leading global health problems, imposing a constant functional and social burden. According to the World Health Organization (WHO), an estimated 5.7% of adults worldwide suffer from depression, with a disproportionately higher prevalence among women (2.3% higher) [1,2]. In this context, antidepressants (ADs) remain the cornerstone of pharmacological management for MDD, particularly in patients with moderate to severe episodes [3]. In routine practice, treatment most often begins with selective serotonin reuptake inhibitors (SSRIs; sertraline or escitalopram) or serotonin–noradrenaline reuptake inhibitors (SNRIs; venlafaxine), tricyclic antidepressants, and so-called “atypical” agents (mirtazapine or vortioxetine) are typically reserved for specific clinical situations, depending on symptom profile, comorbidities, and tolerance considerations [4,5,6].

Despite substantial advances in our understanding of MDD pathophysiology and the development of novel pharmacological agents, several critical unknowns persist, significantly impeding effective patient care. A primary challenge lies in the inherent unpredictability of AD treatment outcomes; a considerable proportion of patients fail to achieve remission with initial therapies, necessitating a protracted and often frustrating «trial-and-error» process [7,8]. This is particularly evident in vulnerable populations, especially for elderly patients, where only about half achieve a 50% reduction in depressive symptoms with antidepressant monotherapy [9]. For cases of severe or treatment-resistant depression, adjunctive interventions like electroconvulsive therapy remain indispensable [3]. Taken together, these observations illustrate how current diagnostic approaches, which aggregate biologically heterogeneous patients under the same MDD label, provide little guidance on which antidepressant (and at what dose) will work for a given individual. This mismatch between the multifactorial, polygenic nature of MDD and the non-personalized approach to prescribing helps explain the suboptimal outcomes seen in routine care. It underscores the need for treatment strategies that explicitly account for inter-individual variability in biological and clinical characteristics.

The conventional monoaminergic hypothesis is insufficient to fully explain the intricate pathophysiology of MDD, driving a paradigm shift towards comprehensive, multi-factorial models incorporating genetic, neuroinflammatory, and neurotrophic factors [10]. This evolving understanding has spurred the development of novel ADs with diverse mechanisms of action (MOA), such as zuranolone (a GABA-A receptor allosteric modulator) and AXS-05, offering new avenues for therapeutic intervention [11,12].

In parallel, advancements in personalized medicine, including therapeutic drug monitoring (TDM) and pharmacogenomic testing (PGT), are gaining traction as promising tools to optimize AD selection and dosing by accounting for individual pharmacokinetic and pharmacodynamic variability [13,14]. Furthermore, the burgeoning fields of Artificial Intelligence (AI) and Machine Learning (ML) are poised to revolutionize psychiatric care by enabling the analysis of vast datasets to identify novel biomarkers, predict treatment response, and facilitate the development of precision diagnostic and therapeutic approaches [15]. This momentum is not unprecedented; AI and ML have already reduced drug development timelines and costs, bringing the per-drug investment from $1.3 billion toward USD 800 million–USD 1.2 billion by accelerating lead discovery, target validation, and pharmacokinetic prediction [16,17,18]. These demonstrated successes in de-risking costly R&D phases provide a compelling precedent for applying similar computational approaches to personalize psychiatric pharmacotherapy of MDD (specifically, by optimizing personalized selection and dosing of ADs), where treatment heterogeneity mirrors the complexity AI/ML has successfully navigated in traditional drug discovery.

However, a critical gap persists in the realization of true precision psychiatry due to the fragmentation of personalized medicine tools, where PGT, TDM, and digital phenotyping are often deployed in isolation. While individual advancements in pharmacogenomics, TDM, and AI/ML offer significant potential, their synergistic application for optimizing personalized treatment remains largely unrealized. The core challenge is not solely technological but lies in synthesizing these disparate data streams (genetic, pharmacokinetic, and real-world behavioral) into a single, clinically actionable prediction model. Key barriers include the methodological and regulatory challenges associated with the necessary validation and standardization of these digital tools, particularly in the context of real-world data (RWD) and real-world evidence (RWE) generation. Addressing the profound heterogeneity of MDD requires a synergistic, multi-modal approach that leverages the computational power of AI/ML to integrate genomic and clinical data, thereby creating algorithms capable of predicting individual treatment response with high fidelity. In this context, the personalization of antidepressant therapy emerges as a logical approach to improving treatment outcomes and addressing the persistent challenge of non-response in MDD.

## 2. Research Objective

*Aim of the study:* The present work is a systematic scoping review conducted to map the available evidence and identify key concepts, sources, and gaps in the literature concerning the integrated application of pharmacogenomics testing (PGT), therapeutic drug monitoring (TDM), and artificial intelligence (AI) in the personalization of antidepressant therapy for Major Depressive Disorder (MDD).

## 3. Global Burden and Socioeconomic Determinants of Major Depressive Disorder

The global burden of Major Depressive Disorder (MDD) is characterized by its pervasive and escalating impact on public health, economies, and social structures worldwide [19]. In 2019, depression was notably ranked as the second-leading cause of disability worldwide; it underscores a substantial contribution to years lived with disability (YLDs) [20]. The economic repercussions are equally severe, with estimates indicating that lost productivity attributable to depression and anxiety collectively imposes an annual cost of about US $1 trillion on the global economy [21]. This immense economic burden highlights the urgent imperative for comprehensive and effective intervention strategies.

Epidemiological trends reveal a dynamic and concerning landscape of MDD prevalence across diverse regions and demographic groups. Between 1990 and 2021, the global prevalence of depressive disorders witnessed a significant increase, with some reports indicating an 88.52% rise in overall depressive disorder prevalence [22]. A more granular analysis focusing specifically on MDD within the same period noted a 56.36% increase globally, with a particularly sharp surge observed among men and young adults aged 20–24 years [23]. While a historical pattern of higher MDD prevalence in females compared to males has been consistently observed across nearly all regions, with age-standardized disability-adjusted life years (DALYs) for females being significantly higher (593) than for males (352) between 1990 and 2019 [24,25], the recent increase in male prevalence in certain age groups indicates a potential narrowing of this long-standing gender gap.

Socioeconomic disparities consistently emerge as critical amplifiers of the MDD burden. Research robustly demonstrates a strong inverse correlation between socioeconomic status (SES) and depression prevalence, with individuals in lower SES strata experiencing disproportionately higher rates of MDD [26,27,28]. For example, a systematic review by Jespersen et al. reported a 70% increase in depression odds among individuals with low composite SES and a 96% increase among those with low income [26]. These gradients reflect a global phenomenon shaped by limited access to healthcare, chronic stress exposure, and reduced availability of support resources.

The profound impact of global stressors on mental health outcomes was starkly illustrated by the COVID-19 pandemic. In its first year, the global prevalence of anxiety and depression surged by a remarkable 25%, highlighting the fragility of mental well-being in the face of widespread crises and emphasizing the critical need for resilient mental health infrastructures [29]. This mental health crisis precipitated a surge in antidepressant prescriptions: global antidepressant consumption increased by 15–20% during 2020–2021, with notable disparities in prescribing patterns across healthcare systems [30,31]. Critically, the urgency of this psychiatric emergency often circumvented rigorous diagnostic and pharmacogenetic assessment, leading to widespread empiric antidepressant initiation with minimal baseline patient characterization or guidance—a pattern particularly pronounced in under-resourced settings and those reliant on “telehealth” models. Consequently, rates of treatment non-response, adverse drug reactions, and treatment discontinuation escalated in tandem with prescription volumes, underscoring a fundamental gap: the absence of precision-based frameworks to match individual patients to optimally efficacious medications. These pandemic-era prescribing patterns (characterized by “trial-and-error” methodology amplified under time pressure) illuminate both the urgency of mental healthcare delivery and the unsustainability of current, non-personalized treatment paradigms.

Although these findings highlight inequities in disease burden, their direct relevance to antidepressant therapy lies in the fact that socioeconomic disadvantage often restricts access to timely diagnosis, appropriate medication selection, and follow-up care. This variability in access and treatment quality underscores the broader challenge of delivering consistent and effective antidepressant therapy across populations and further supports the need for personalized, data-informed approaches.

### 3.1. Understanding of MDD Pathophysiology

**Figure 1:**
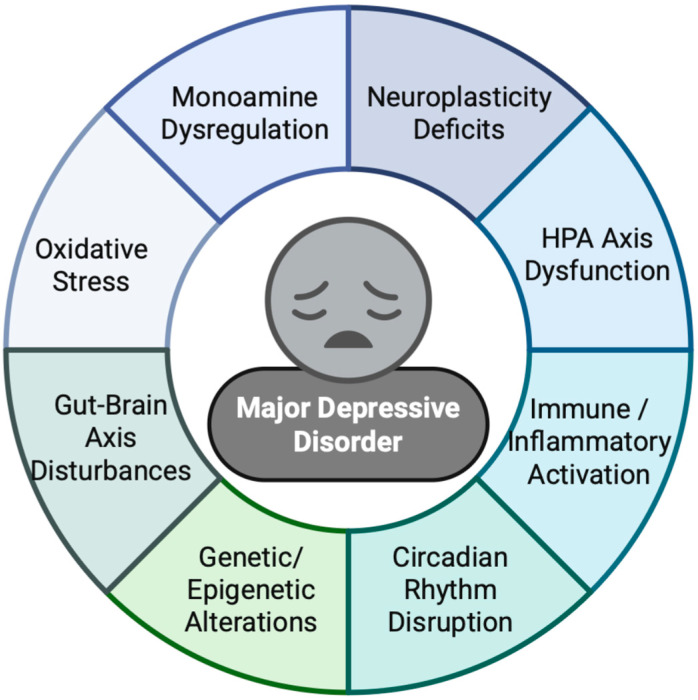
Schematic representation of the core pathophysiological axes underlying Major Depressive Disorder (MDD).

Major Depressive Disorder (MDD) is a complex and multifactorial psychiatric condition, the pathogenesis of which extends beyond simplistic explanations to encompass a sophisticated interplay of physiological, biochemical, genetic, and molecular mechanisms (Figure 1) [32]. Recent advancements in neuroscience have elucidated several key pathways and hypotheses that collectively contribute to our evolving understanding of this debilitating disorder.

At its core, MDD involves profound alterations in neurobiological systems. The traditional monoamine hypothesis, while foundational, has evolved from a simple theory of neurotransmitter depletion to a more nuanced understanding of receptor dynamics and broader neuroregulatory systems. Initial observations suggested that reduced levels of serotonin (5-HT), noradrenaline (NE), and dopamine (DA) in the central nervous system were central to depression [33]. However, the delayed therapeutic onset of selective serotonin reuptake inhibitors (SSRIs) and serotonin-noradrenaline reuptake inhibitors (SNRIs), despite acute increases in synaptic monoamine concentrations, highlighted the limitations of this singular view [34,35]. This simplistic deficiency model is now largely considered a historical oversimplification, lacking empirical support for a direct causal link between low serotonin levels and MDD [36,37]. From a clinical pharmacology perspective, the notion of a ‘serotonin dosage exam’ is scientifically unsound, as peripheral monoamine levels do not reliably reflect central nervous system concentrations, and the therapeutic effect of ADs is not solely dependent on acute synaptic availability [38]. Instead, the efficacy of SSRIs and SNRIs is now understood to be mediated by downstream neuroplastic changes, including the upregulation of neurotrophic factors and altered receptor sensitivity, which take weeks to manifest [39]. Furthermore, the significant inter-individual variability in antidepressant response is heavily influenced by pharmacokinetics (e.g., CYP450 enzyme polymorphisms) and pharmacodynamics (e.g., receptor binding affinity), factors entirely independent of a baseline “serotonin deficit” [40].

**Figure 2:**
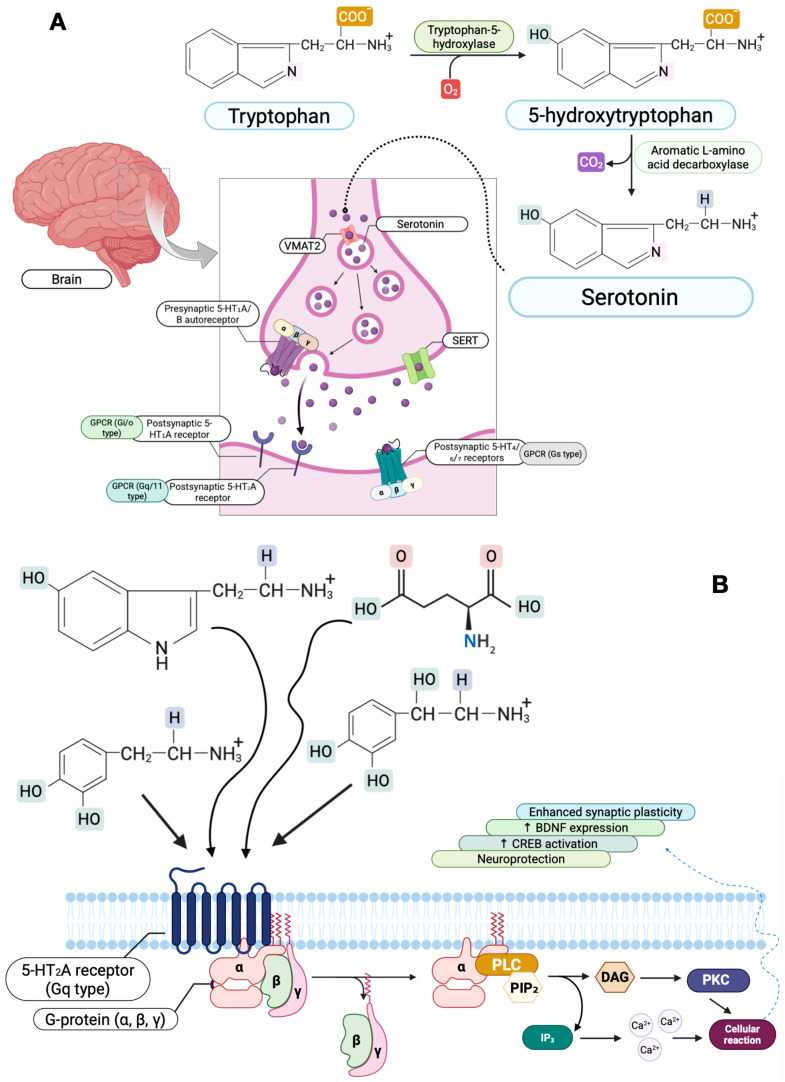
Serotonin biosynthesis and Gq-coupled signaling cascades implicated in Major Depressive Disorder (MDD). (**A**) Serotonin biosynthesis and synaptic transmission: tryptophan, an essential amino acid, is converted to 5-hydroxytryptophan (5-HTP) by tryptophan-5-hydroxylase (TPH) and further decarboxylated by aromatic L-amino acid decarboxylase (AADC) to yield serotonin (5-hydroxytryptamine, 5-HT). Newly synthesized 5-HT is stored in presynaptic vesicles via the vesicular monoamine transporter 2 (VMAT2) and released into the synaptic cleft. Serotonin modulates neurotransmission by binding to multiple receptor subtypes: presynaptic 5-HT1A/B autoreceptors (negative feedback on release) and postsynaptic Gi/0-, Gq/11-, and Gs-coupled receptors (e.g., 5-HT1A, 5-HT2A, 5-HT4/6/7). Clearance of 5-HT from the synaptic cleft occurs through the serotonin transporter (SERT); dysregulation of this synthesis-release-reuptake axis contributes to serotonergic deficiency and impaired mood regulation observed in MDD. (**B**) Post-synaptic Gq-coupled 5-HT2A signaling and neuroplastic outcomes: activation of Gq-coupled 5-HT2A receptors (by initiates a signaling cascade through G-protein (Gα(q), Gβ, Gγ) activation of phospholipase C-β (PLC(β)), which hydrolyses phosphatidylinositol-4,5-bisphosphate (PIP2) into diacylglycerol (DAG) and inositol-1,4,5-trisphosphate (IP3). IP3 stimulates Ca2+ release from endoplasmic reticulum stores via IP3 receptors (IP3R), while DAG and Ca2+ jointly activate protein kinase C (PKC). The rise in intracellular Ca2+ also activates calmodulin (CaM) and Ca2+/calmodulin-dependent kinase II (CaMK-II). Both PKC and CaMKII converge on the ERK1/2 (MAPK) pathway, promoting phosphorylation of CREB (cAMP-response element-binding protein) and subsequent transcription of BDNF (brain-derived neurotrophic factor). This pathway enhances synaptic plasticity, neuroprotection, and resilience, mechanisms often compromised in MDD and restored by antidepressant therapy.

Current perspectives emphasize the monoamine receptor hypothesis, positing that an imbalance in monoamine receptor activation, rather than mere depletion, underlies MDD. The complexity of this signaling is now understood through the lens of G protein-coupled receptor (GPCR) dynamics, where concepts as allosteric modulation and biased agonism dictate specific intracellular outcomes, moving beyond simple receptor occupancy to pathway-selective activation [41]. For instance, the 5-HT1A receptors, which are inhibitory and Gi-coupled, play a role in stress tolerance within limbic structures, while excitatory, Gq-coupled 5-HT2A receptors in the cortex are implicated in plasticity and environmental sensitivity [42,43]. The differential modulation of these receptors by conventional antidepressants and novel agents like psychedelics underscores the intricate nature of serotonergic signaling in mood regulation (Figure 2).

But the failure of the monoamine theory to explain the delayed response to classic ADs has spurred a paradigm shift toward neuroplasticity and the glutamatergic system. The discovery of rapid-acting antidepressants like ketamine, which primarily target N-methyl-D-aspartate receptors (NMDARs), has revealed that the ultimate therapeutic effect is mediated by the rapid induction of synaptic plasticity, specifically the formation of new dendritic spines via the mTOR signaling pathway [44]. This mechanism underscores a broader neurotrophic dimension to MDD, where chronic stress and depression are associated with a loss of synaptic integrity and an imbalance between pro-survival factors (like mature BDNF, brain-derived neurotrophic factor) and pro-degenerative signaling pathways (e.g., via p75 neurotrophin receptor) [45]. Thus, effective antidepressant action is increasingly viewed as the restoration of neuronal resilience and synaptic connectivity, a process that requires a system-level understanding far exceeding the initial monoamine deficit theory.

Beyond monoamines, the glutamatergic and GABAergic systems are increasingly recognized as critical players in MDD pathophysiology [46,47]. Abnormal connectivity and dysregulation within these excitatory and inhibitory neurotransmitter systems contribute significantly to the neural circuit dysfunction observed in depression [48]. The involvement of these pathways suggests a broader synaptic imbalance that affects neuronal excitability and communication throughout the brain.

Neurotrophic factors, particularly brain-derived neurotrophic factor (BDNF), represent another pivotal area of investigation. The neurotrophin hypothesis of depression posits that reduced hippocampal BDNF levels are strongly associated with stress-induced depressive states [49,50]. BDNF is crucial for synaptic plasticity, neuronal differentiation, maintenance, outgrowth, and repair. Evidence from postmortem studies and animal models indicates that BDNF depletion impairs neurogenesis, a process vital for brain health and function, thereby contributing to the onset and progression of MDD. Antidepressant treatments are thought to exert their therapeutic effects, in part, by normalizing BDNF levels in the brain, highlighting a potential mechanism for neuroplastic recovery.

**Figure 3:**
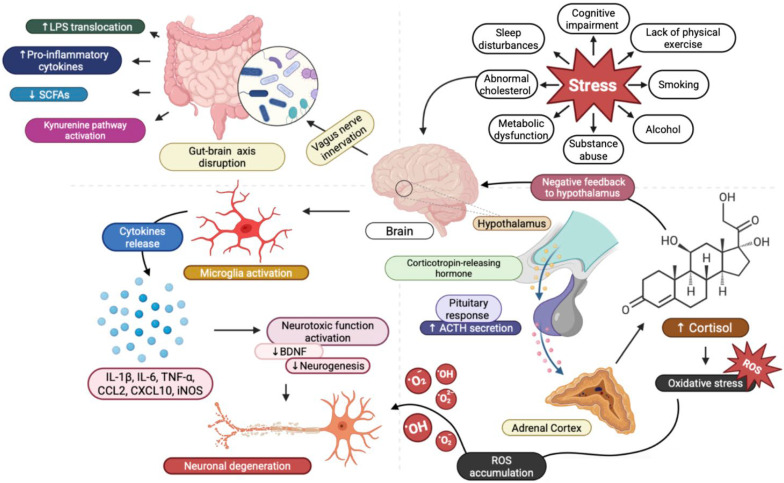
Interconnected pathways linking stress, «gut–brain» axis disruption, neuroinflammation, and oxidative stress in Major Depressive Disorder (MDD). Stress induced by environmental, metabolic, or behavioral factors activates central stress circuitry in the brain, triggering both neuroendocrine and immune responses. Chronic stress contributes to dysregulation of the hypothalamic–pituitary–adrenal (HPA) axis, characterized by increased release of corticotropin-releasing hormone (CRH) from the hypothalamus and adrenocorticotropic hormone (ACTH) from the pituitary, leading to sustained cortisol elevation. Excess cortisol promotes glucocorticoid receptor (GR) resistance, mitochondrial dysfunction, and accumulation of reactive oxygen species (ROS), amplifying oxidative stress. Simultaneously, stress-induced alterations in gut permeability and microbiota composition disrupt the «gut–brain» axis, resulting in lipopolysaccharide (LPS) translocation, activation of the kynurenine pathway, and release of pro-inflammatory cytokines: interleukin-1β (IL-1β), interleukin-6 (IL-6), and tumor necrosis factor-α (TNF-α). These cytokines activate microglia, which secrete additional inflammatory mediators (e.g., CCL2, CXCL10, inducible nitric oxide synthase—iNOS), reduce brain-derived neurotrophic factor (BDNF) expression, and impair neurogenesis.

In parallel, inflammation and immune dysregulation have emerged as significant contributors to MDD. The cytokine hypothesis suggests that elevated levels of pro-inflammatory cytokines can induce depressive symptoms by affecting neurotransmitter metabolism, neurogenesis, and neuroendocrine function [51]. This neuroinflammatory state is fundamentally linked to a shift in microglial phenotype and is inseparable from the concept of oxidative stress. Oxidative stress, defined as an imbalance between reactive oxygen species (ROS) production and the capacity for antioxidant defense, acts synergistically with chronic inflammation to drive MDD pathogenesis [52]. Specifically, pro-inflammatory cytokines, while including the classic mediators (IL-1β, IL-6, TNF-α), exert their neurotoxic effects by impairing mitochondrial function and activating the kynurenine pathway (KYN). Activation of the KYN pathway, mediated by the enzyme indoleamine 2,3-dioxygenase (IDO), shunts tryptophan metabolism away from the synthesis of serotonin and toward the production of neurotoxic KYN metabolites (e.g., quinolinic acid), thereby providing a direct mechanistic link between immune activation and monoamine depletion. In addition, neuroinflammation is often linked to epigenetic modifications, which can alter gene expression without changing the underlying DNA sequence [53]. Epigenetic factors, as DNA methylation, histone modification, and microRNA expression, can mediate neuroinflammatory changes and influence susceptibility to MDD [54,55]. The interplay between genetic predispositions, environmental stressors, oxidative stress, and inflammatory responses creates a complex web of interactions that shapes an individual’s vulnerability to depression (Figure 3).

The hypothalamic–pituitary–adrenal (HPA) axis dysfunction is a well-established component of MDD pathophysiology. Chronic stress can lead to HPA axis hyperactivity, resulting in elevated glucocorticoid levels [56,57,58]. However, accumulating evidence shows that patients with MDD may also present with normocortisolemia or even hypocortisolemia, reflecting distinct patterns of HPA feedback dysregulation rather than a uniform hyperactive state. These divergent cortisol profiles are clinically relevant, as persistent hypercortisolemia has been associated with reduced antidepressant response and higher relapse risk, whereas more normalized cortisol dynamics tend to accompany better therapeutic outcomes [59,60]. While glucocorticoids are essential for stress response, their prolonged elevation can lead to neurotoxicity, particularly in the hippocampus, and contribute to the structural and functional brain remodeling observed in MDD [61,62]. Postmortem studies of MDD patients have revealed reduced densities of glial cells, particularly astrocytes, in key brain regions such as the prefrontal cortex (PFC), hippocampus, and amygdala [63,64,65]. Astrocytic dysfunction, characterized by altered levels of glial fibrillary acidic protein (GFAP), connexins, glutamine synthase (GS), glutamate transporter-1 (GLT-1), and aquaporin-4 (AQP4), plays a substantial role in the pathophysiology, affecting neurotransmitter homeostasis and neuronal support [66,67]. Importantly, some antidepressants appear to exert indirect pro-glial or neurotrophic effects, suggesting that glial health and HPA axis status may contribute to interindividual variability in treatment response [68].

From a morphological perspective, MDD is associated with observable structural changes in the brain. These include volumetric reductions in the hippocampus and other forebrain regions, as well as alterations in neuronal and glial cell morphology and density [69,70].

### 3.2. Multifactorial Variability in the Choice of Antidepressant

The clinical reality of antidepressant therapy is defined not merely by drug class, but by the profound, multi-factorial variability inherent to specific agents. This therapeutic uncertainty stems from a complex interplay of genetic and non-genetic factors that dictate drug disposition and receptor engagement.

For example, the metabolism of most widely prescribed antidepressants is critically dependent on the highly polymorphic Cytochrome P450 (CYP450) enzyme system. For instance, the SSRIs fluoxetine and paroxetine are potent inhibitors of CYP2D6, creating a high risk for clinically significant drug–drug interactions, while escitalopram relies heavily on CYP2C19 [38,69]. Similarly, TCAs like amitriptyline and nortriptyline are extensively metabolized by both CYP2D6 and CYP2C19, and their narrow therapeutic index means that minor variations in a patient’s CYP450 genotype can lead to wildly divergent plasma concentrations, resulting in either therapeutic failure (in ultra-rapid metabolizers) or cardiotoxicity (in poor metabolizers) [70]. This metabolic heterogeneity, which also affects SNRIs as venlafaxine (a CYP2D6 substrate), directly impacts the ratio of parent drug to active metabolite, highlighting the limitations of fixed-dose prescribing [71].

On the other hand, beyond pharmacogenetics, the unpredictability of antidepressant response is further compounded by critical non-genetic factors. Age significantly impacts both pharmacokinetics (due to changes in hepatic and renal function) and pharmacodynamics, with response patterns differing substantially between adolescent, adult, and geriatric populations [72,73]. Sex differences influence drug metabolism and efficacy, often mediated by hormonal fluctuations and differential enzyme activity [74]. Furthermore, comorbidity (both psychiatric and physical) and the underlying neuroinflammatory state of the patient are now recognized as powerful modulators of treatment outcome, with elevated pro-inflammatory markers often correlating with reduced antidepressant efficacy [75,76].

Ultimately, the clinical challenge lies in the unpredictability of individual response driven by the convergence of these specific genetic and non-genetic differences. This multifactorial therapeutic uncertainty forms the basis for the integration of PGT, TDM, and AI/ML in order to go beyond the existing empirical paradigm.

## 4. Advancement in Personalized Medicine for MDD

### 4.1. Therapeutic Drug Monitoring (TDM)

Therapeutic drug monitoring (TDM) is a tool for measuring drug concentrations in biological fluids. While most reference values are developed for standard matrices, such as plasma and serum, antidepressants are usually measured in whole blood for determining therapeutic, toxic, and lethal doses [77]. In addition, TDM allows differences in the pharmacokinetic (PK) (what the body does to the drug) and pharmacodynamic (PD) (what the drug does to the body) parameters of different classes of drugs to be identified through the use of individual measurements to optimize drug dosing for each patient.

Therapeutic drug monitoring is relevant in the general intensive care unit (ICU), cardiology, surgery ICU, and pediatrics departments [78]. Drugs for which modern literature recommends TDM have a narrow therapeutic window, high interindividual variability in PK/PD, large variation in metabolic pathways, long duration of therapy, and severe consequences of overdose. However, the principles underpinning TDM (for example, high inter-individual pharmacokinetic (PK) variability, the potential for severe adverse effects, and the critical need to establish a concentration-effect relationship) are profoundly relevant to the field of psychopharmacology. In antidepressant therapy, these same principles translate directly into the challenge of predicting clinical response based on dose alone, as nominally identical regimens can yield markedly different drug exposures across patients [79].

Thus, even though antidepressants are not routinely monitored in the ICU setting, the pharmacological rationale supporting TDM in critical-care environments is directly transferable to MDD management: large PK variability, narrow therapeutic windows for certain drug classes (notably TCAs), and a substantial burden of adverse events all justify individualized concentration-guided dosing [80,81].

Antidepressant therapy is plagued by a high rate of non-response and the frequent occurrence of dose-limiting side effects, both of which are often attributable to unpredictable drug exposure [82]. For psychotropic agents, PK variability is not just a statistical parameter but a clinical predictive indicator, as a standard dose can result in sub-therapeutic concentrations in one patient and toxic levels in another [83]. This variability is driven by a complex interplay of genetic polymorphisms (e.g., in CYP450 enzymes), physiological factors, and drug–drug interactions. Therefore, TDM for antidepressants serves a dual purpose: to ensure adequate exposure for therapeutic efficacy and to mitigate the risk of toxicity, making it an essential tool for advancing personalized medicine in MDD. Importantly, when interpreted alongside pharmacogenetic results (e.g., CYP2C19 or CYP2D6 genotypes), TDM can bridge the gap between predicted and actual drug exposure, revealing whether the observed concentration aligns with expected metabolic capacity [84]. Furthermore, incorporating TDM data into model-informed precision dosing algorithms or machine-learning systems could enable real-time dose adjustment, prediction of treatment response, and early identification of patients at risk of non-response or adverse events, thereby operationalizing TDM as a central component of truly individualized antidepressant therapy.

Population pharmacokinetic methods (popPK) are used in TDM to identify differences between drug classes in pharmacokinetic parameters that characterize the main pharmacokinetic processes (absorption, distribution, metabolism (biotransformation), and excretion) [85,86]. The absorption rate (the rate at which the drug enters the systemic circulation from the site of administration in the case of non-intravenous administration) determines the absorption processes [87]. The volume of distribution (Vd) (which reflects the proportion of the substance remaining in the extravascular space) and the distribution rate (the rate at which equilibrium between the concentration of the drug in the blood and tissue is reached) characterize distribution [88]. Bioavailability (F) (the amount of drug that enters the systemic circulation; the AUC (area under the curve) parameter is used to calculate F)—metabolism [89]. Excretion processes are reflected in the half-life (T1/2) (the time it takes for the concentration of a substance in serum/the body to decrease by half), total clearance (reflects the volume of biological fluid that is cleared of a given substance per unit of time by the organs involved in excretion—the liver, kidneys, glands) [90].

However, for antidepressants, these parameters acquire clinical significance only when interpreted at the individual-patient level. While population PK models quantify typical ranges for Vd, clearance, and AUC, the true therapeutic value arises when deviations from these expected ranges are used to guide treatment adjustments. In practice, popPK acts as the mathematical backbone for individualized dosing algorithms: it allows clinicians to estimate whether a patient’s measured antidepressant concentration is explainable by known factors (e.g., CYP2D6 status, renal function, age, or body composition) or whether an unexpected outlier reflects non-adherence, drug–drug interactions, or an atypical metabolic phenotype.

For example, Yoonhyuk Jang and co-authors developed popPK for oxazepine (an antiepileptic drug) to identify undesirable dose-dependent effects based on apparent clearance, apparent volume of distribution, and AUC [91]. A similar modeling approach is increasingly used for antidepressants, where popPK-guided simulations can predict whether dose escalation will safely achieve therapeutic exposure or push the patient toward toxicity, particularly relevant for TCAs, venlafaxine, duloxetine, or escitalopram.

**Figure 4:**
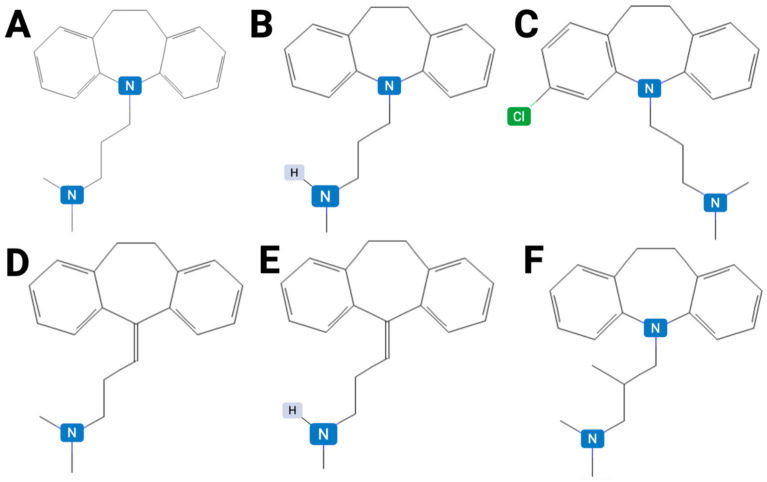
Chemical structures of representative tricyclic antidepressants (TCAs) used in the treatment of major depressive disorder (MDD): (**A**) imipramine (IUPAC name: 3-(5,6-dihydrobenzo[b][1]benzazepin-11-yl)-N,N-dimethylpropan-1-amine), (**B**) desipramine (IUPAC name: 3-(5,6-dihydrobenzo[b][1]benzazepin-11-yl)-N-methylpropan-1-amine), (**C**) clomipramine (IUPAC name: 3-(2-chloro-5,6-dihydrobenzo[b][1]benzazepin-11-yl)-N,N-dimethylpropan-1-amine), (**D**) amitriptyline (IUPAC name: N,N-dimethyl-3-(2-tricyclo[9.4.0.03,8]pentadeca-1(15),3,5,7,11,13-hexaenylidene)propan-1-amine), (**E**) nortriptyline (IUPAC name: N-methyl-3-(2-tricyclo[9.4.0.03,8]pentadeca-1(15),3,5,7,11,13-hexaenylidene)propan-1-amine), (**F**) trimipramine (IUPAC name: 3-(5,6-dihydrobenzo[b][1]benzazepin-11-yl)-N,N,2-trimethylpropan-1-amine).

For tricyclic antidepressants (TCAs), popPK studies were instrumental in quantifying the profound inter-individual variability in clearance (CL) and volume of distribution (Vd), thereby solidifying the necessity of TDM due to their narrow therapeutic index (Figure 4) [92]. The immediate clinical integration of TDM for TCAs is fairly well described in the medical literature. Studies consistently demonstrate a clear concentration-effect relationship, especially for nortriptyline, where a therapeutic reference range (TRR) of 50–150 ng/mL is widely accepted [93]. The high interindividual variability in TCA metabolism, largely determined by CYP2D6 and CYP2C19 polymorphism, makes TDM an important tool for ensuring safety and efficacy.

TDM results for TCAs can be directly translated into individualized dosing strategies: measured concentrations enable clinicians to adjust the dose with mathematical precision, rather than relying on symptom-guided titration alone [94]. In practice, integrating TDM with pharmacogenetic results (e.g., confirming CYP2D6 poor metabolizer status through elevated parent/metabolite ratios) allows identification of patients who require dose reductions, alternative agents, or slower titration schedules. This concentration-guided approaches form the basis for model-informed precision dosing systems, which are increasingly applied to TCAs due to their narrow therapeutic index.

For newer antidepressants like SSRIs (for example, citalopram, sertraline) and SNRIs (venlafaxine), TDM is generally considered optional but highly recommended in specific clinical scenarios. Although these drugs possess a wider therapeutic index than TCAs, significant PK variability still exists, leading to non-response or intolerable side effects. For instance, studies on venlafaxine and its active metabolite O-desmethylvenlafaxine have shown that patients with low plasma concentrations are significantly less likely to achieve remission [95,96]. Furthermore, the clinical relevance of TDM is heightened for drugs with known genetic variability, such as citalopram and escitalopram, whose metabolism is heavily influenced by CYP2C19 and CYP2D6, leading to up to 40-fold concentration differences between poor and ultrarapid metabolizers [97]. In these cases, TDM acts as a tool, validating the functional metabolic status predicted by pharmacogenomic testing.

When applied longitudinally, TDM helps determine whether inadequate response reflects true pharmacodynamic resistance or simply insufficient drug exposure. For agents with active metabolites (e.g., venlafaxine to ODV), metabolite ratios can further guide individualized treatment, distinguishing between rapid metabolizers who may require higher doses and patients with metabolic saturation who benefit from switching medications rather than dose escalation [98]. The individualized interpretation is increasingly incorporated into clinical decision-support tools, where TDM values serve as quantitative inputs for algorithms that estimate the probability of remission at a given concentration.

However, it is also important to discuss that the lack of universally accepted therapeutic plasma concentration reference ranges for all SSRIs and SNRIs remains a critical barrier to their routine TDM application. However, the emerging consensus is that TDM is indispensable for complex cases, including treatment-resistant depression, non-response, severe side effects, polypharmacy, and suspected non-adherence, effectively transforming TDM from a routine test into a precision diagnostic tool [99].

Thus, popPK-informed TDM not only quantifies exposure but also clarifies the mechanistic reason for non-response or side effects, enabling rational dose adjustment or drug substitution tailored to the individual patient. Crucially, these PK-derived metrics can be fed into model-informed precision dosing (MIPD) platforms [100], where Bayesian forecasting uses TDM values to simulate individualized concentration–time profiles and recommend personalized dosing regimens [101,102]. This integration moves TDM beyond a descriptive tool and positions it as an operational component of personalized antidepressant therapy, particularly when combined with pharmacogenetic status and real-world patient characteristics.

### 4.2. Analytical Methods and Clinical Imperative

The analytical foundation of TDM has long rested on highly specific and sensitive chromatographic methods. High-performance liquid chromatography (HPLC) coupled with mass spectrometry (MS/MS) remains the gold standard, offering high specificity and sensitivity for simultaneous determination of multiple antidepressants and their active metabolites in various biological samples (e.g., urine, blood, or saliva) [103,104]. Sample preparation (it is necessary to obtain reliable analysis results) typically involves liquid–liquid extraction or solid-phase sorption, with recent studies exploring «green» methods like reverse micelle-assisted dispersive liquid–liquid microextraction (RM-DLLME) and microextraction using nanoparticles (SUPRAS) [105,106]. But their accuracy requires costly equipment and labor-intensive sample preparation (and, of course, there is a great need for trained staff), creating a critical time gap between dose administration and therapeutic assessment [107].

To overcome this limitation, the field is rapidly pivoting toward on-site monitoring technologies. Biosensors, which convert a biological recognition event into a measurable signal, offer the potential for rapid, cost-effective, and decentralized TDM [108]. These devices, utilizing electrochemical or optical transducers, enable near-continuous data capture, a crucial step toward achieving true model-informed precision dosing.

We hypothesize that the future of TDM for antidepressants lies not in the analytical measurement itself, but in the predictive modeling of the concentration-effect relationship, a task uniquely suited for artificial intelligence (AI) and machine learning (ML).

**Figure 5:**
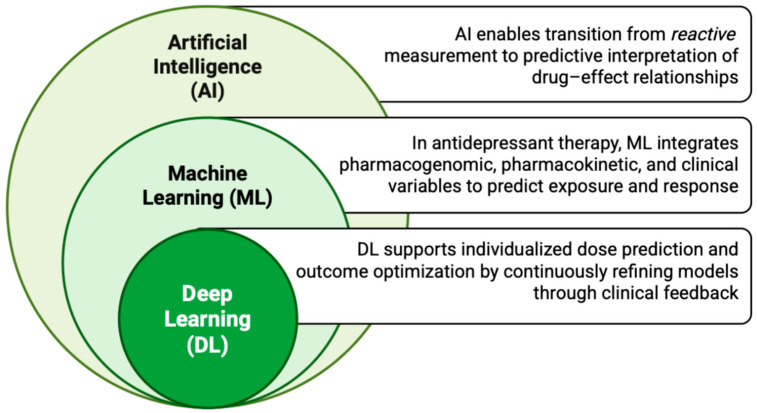
Conceptual framework for integrating artificial intelligence (AI), machine learning (ML), and deep learning (DL) into predictive therapeutic drug monitoring (TDM) for antidepressants.

Traditional TDM is fundamentally reactive, providing a snapshot of drug exposure after the fact of intervention. However, the integration of AI could transform this process into a proactive, predictive tool (Figure 5) [109]. ML algorithms excel at identifying non-linear and complex correlations between high-dimensional input data: pharmacogenomic profiles (for example, CYP2D6/2C19 metabolizer status), clinical covariates (age, BMI, comedications), and TDM values—and the ultimate clinical outcome (response/remission/side effects) [110,111]. This is particularly critical in psychopharmacology, where the therapeutic window is often defined by a subtle balance between efficacy and central nervous system-related side effects, a balance that is highly individualized.

The critical advantage of AI is its ability to move beyond the limitations of classical population pharmacokinetic models, which rely on predefined structural equations. Instead, AI models can construct a dynamic, patient-specific digital twin that continuously learns from real-world data (RWD) and real-time TDM measurements (potentially from biosensors) [112]. This digital twin allows for the prediction of the optimal dose before the first dose is administered and provides continuous, model-informed precision dosing adjustments.

By leveraging AI to quantify the relative contribution of genetic factors (PGT) versus environmental and physiological factors to PK variability, the field can finally transition from the persistent trial-and-error approach in MDD management to a truly personalized, predictive, and optimized therapeutic strategy.

## 5. Pharmacogenetics Testing for Antidepressant Response

As discussed in the section “Global burden and socioeconomic determinants of major depressive disorder”, MDD has a heterogeneous pathogenesis, which requires a comprehensive approach to provide reliable information about the disease. To date, studies using both candidate gene (pharmacogenetics (PGx)) and whole-genome (pharmacogenomic) approaches have successfully identified genetic variations associated with treatment outcomes in psychiatry.

Knowledge of genetic polymorphisms contributes to the discovery of target genes that determine pharmacokinetics (absorption, distribution, metabolism, and excretion) [91] and pharmacodynamics (receptors, ion channels, enzymes, and immune system) in the responses of patients undergoing therapy. In addition, studies show that 15% to 30% of the variability in response to treatment is due to genetic factors [113,114,115]. Identifying specific genetic variants can therefore help predict individual drug responses and adverse effects, enabling improved treatment outcomes through personalized therapy.

Cytochrome P450 enzymes play a key role in the biotransformation of antidepressants in the body, forming key metabolic pathways and determining the effectiveness of therapy [110]. Variations in the genes of isomorphic forms of P450 alter the metabolic activity of the enzyme itself, ranking patients from poor to ultra-rapid metabolisers, which clinicians must take into account when selecting a drug dosage [116]. For example, Hanna Maria Kariis et al. confirmed in their study that a decrease in CYP2C19 enzyme activity increases the concentration of antidepressants in blood plasma and vice versa (CYP2C19 poor metabolisers have increased odds of side effects with CYP2C19-metabolized antidepressants, while ultrarapid metabolisers have lower odds of side effects) [117]. Thus, genotyping of P450 genetic variations can be used to prevent side effects due to high plasma drug concentrations, as well as treatment failure due to low concentrations [118].

These pharmacokinetic differences cannot be reliably inferred from clinical symptoms alone; therefore, integrating pharmacogenetic information with early therapeutic drug monitoring (TDM) provides an objective way to confirm whether the measured concentration corresponds to the predicted metabolic phenotype. This is particularly relevant for antidepressants with CYP-dependent metabolism, as citalopram, escitalopram, sertraline (CYP2C19), as well as venlafaxine, paroxetine, fluoxetine, and nortriptyline (CYP2D6), where genotype-based predictions often require verification through measured plasma levels [119,120,121,122]. When combined, PGT and TDM allow clinicians to distinguish between true metabolic variability and confounding factors such as non-adherence, drug–drug interactions, or unexpected accumulation, thereby enabling precise dose individualization [123]. In practice, confirming whether a CYP2C19 poor metabolizer on escitalopram has supratherapeutic exposure, or whether a CYP2D6 ultrarapid metabolizer on venlafaxine has subtherapeutic concentrations, directly informs whether the clinician should reduce, escalate, or switch therapy. Furthermore, CYP-based variability represents a key input for machine-learning or decision-support algorithms, which can incorporate genotype, measured plasma levels, and clinical features to forecast optimal dosing trajectories and identify patients at risk of non-response or adverse effects [124,125].

In addition to genetic variations in P450 (which affect pharmacokinetic parameters), pharmacodynamically relevant genes (genes encoding transporter proteins and immune system cells) also influence the efficacy of antidepressants. For example, Brunoni et al. demonstrated that variations in the SLC6A4 gene, particularly the 5-HTTLPR polymorphism, are associated with differential SSRI efficacy and tolerability [98]. Moreover, immunologically relevant genes (encoding the human leukocyte antigen) have an influence. The human leukocyte antigen can form complexes with certain drugs, which will be perceived as foreign by the immune system [126].

These pharmacokinetic and pharmacodynamic factors frequently converge in clinical practice, illustrating the need for truly individualized therapy. For instance, consider a patient with CYP2C19 poor-metabolizer status who initiates citalopram. Pharmacogenetic testing correctly predicts reduced metabolic capacity, and subsequent therapeutic drug monitoring (TDM) confirms elevated plasma concentrations despite a standard dose. A clinical decision-support tool integrating PGT and TDM data recommends a lower starting dose or a switch to a CYP2C19-independent SSRI, for example, sertraline, thereby reducing the likelihood of adverse effects while preserving therapeutic efficacy.

A second scenario highlights the importance of pharmacodynamic variation. A patient carrying the SLC6A4 short (s)-allele initiates escitalopram but reports minimal improvement at week four. TDM demonstrates adequate plasma exposure, ruling out underdosing, while digital phenotyping from a wearable device shows persistent sleep fragmentation and reduced activity, a pattern often associated with SSRI non-response in s-allele carriers. An AI-based clinical algorithm synthesizes these data (PGT + TDM + behavioral signals) and suggests switching to a noradrenergic or multimodal agent such as venlafaxine or vortioxetine, which may offer improved efficacy in this genetic and behavioral context. Together, these examples illustrate how pharmacokinetic genes (e.g., CYP2C19), pharmacodynamic markers (e.g., SLC6A4), TDM, and digital phenotyping can be operationalized to tailor antidepressant therapy to the individual, fulfilling the principles of precision medicine.

The clinical utility of PGT-guided prescribing has been validated in several large-scale studies. For example, a meta-analysis by Li et al., using genotype imputation from 13 clinical studies, confirmed the significant influence of CYP2C19 and CYP2D6 metabolic activity on antidepressant response, providing robust evidence for the PK-PGT link [127]. More recently, the Alexander et al. systematic review highlighted that PGT-guided antidepressant therapy led to faster initial remission and response in patients with MDD, demonstrating a persistent benefit over 6 months compared to treatment as usual [97]. Clinically, these findings translate into actionable decisions for commonly used agents as escitalopram, citalopram, sertraline, paroxetine, venlafaxine, and amitriptyline, where genotype-driven dose modifications or drug substitutions can meaningfully reduce adverse effects and improve therapeutic engagement. However, most commercial PGT panels predominantly examine single-gene variants, which limits predictive power in a polygenic disorder like MDD, where metabolic (PK) genes interact with receptor, transporter, and immunogenetic (PD) pathways. As a result, there is increasing scientific momentum toward incorporating polygenic predictors, integrating multiple PK and PD loci, to better model the multilevel biological heterogeneity that influences antidepressant outcomes [128,129].

The inherent complexity of PGT data: comprising thousands of SNPs, copy-number variations, gene–gene interactions, and their dependence on clinical and environmental factors, makes traditional linear prediction models insufficient for therapy optimization [130]. This gap defines the critical role of artificial intelligence AI and ML as computational accelerators in precision psychopharmacology.

The application of machine learning to personalized medicine is not a nascent concept, but rather one that has evolved significantly from foundational work in the early 2010s. These early studies established the feasibility of using computational models to integrate complex, multi-modal data for predictive pharmacotherapy, a principle directly relevant to the personalization of antidepressant treatment.

One of the earliest and most influential examples comes from the field of oncology, where Menden et al. developed machine learning models (specifically, neural networks) to predict the sensitivity of cancer cell lines to various drugs [131]. This work was pioneering in its integration of two distinct data streams: genomic features (mutation status of 77 oncogenes) and chemical properties of the drugs themselves. The ability to predict drug response (quantified as IC50 values) with high accuracy (R^2^ of 0.72 in cross-validation) demonstrated the power of ML to bridge molecular and pharmacological data, laying the groundwork for similar multi-omic approaches in psychiatry.

In parallel, Kessler et al., utilizing data from the World Mental Health surveys, demonstrated that ML algorithms could significantly improve the prediction of MDD persistence, chronicity, and severity over conventional logistic regression models [132]. By analyzing baseline self-report data (phenotypic and clinical variables), the ML models achieved superior accuracy (AUC up to 0.76), proving that complex clinical outcomes could be reliably predicted from readily available patient data.

A key challenge is the integration of pharmacogenomic data, which often involves complex interactions between multiple genes (polygenic effects). Taliaz et al. addressed this by developing an ML model that integrated genetic, clinical, and demographic data to predict response to a panel of antidepressants. Their model, which utilized a proprietary polygenic algorithm, outperformed models based on clinical or genetic data alone. This highlights the necessity of a multi-modal approach, where AI/ML serves as the essential computational engine for synthesizing disparate data streams into a single, clinically actionable prediction [133].

Further solidifying this trend, Bobo et al. systematically reviewed the integration of ML and pharmacogenomics for predicting antidepressant response, concluding that ML methods utilizing pharmacogenomic and clinical data show promising results for predicting short-term response [134]. More recently, the focus has shifted toward clinical translation through the development of clinical decision support systems (CDSS). Benrimoh et al. are currently conducting a cluster randomized trial (AID-ME) to evaluate a deep-learning-enabled CDSS for depression medication enhancement [135].

AI algorithms can simultaneously integrate pharmacogenetic data, early therapeutic drug monitoring (TDM) measurements, and digital phenotyping signals (e.g., sleep, circadian patterns, heart-rate variability, physical activity) collected through smartphones or wearables, generating individualized response forecasts that cannot be achieved by isolated data streams [136,137]. By leveraging deep learning architectures, researchers can move beyond CPIC-style single-gene guidance and develop multivariate models that identify hidden non-linear patterns linking genotype, exposure, and symptom trajectories [138,139].

Recent AI-based frameworks in psychiatric pharmacotherapy have shown that combining CYP2C19/CYP2D6 genotype with plasma levels of escitalopram or venlafaxine improves prediction accuracy for early treatment response, while ML classifiers incorporating digital-behavioral markers enhance detection of emerging non-response or adverse effects [140,141,142]. Collectively, these integrative approaches illustrate how AI enables the synthesis of PK genes, PD targets, environmental context, adherence patterns, and real-world physiological data into a unified decision-support system—aligning directly with the concept of truly personalized medicine.

## 6. Future Directions and Conclusions

The persistent challenge of major depressive disorder MDD management, characterized by a high rate of non-response and the inherent unpredictability of empirical antidepressant selection, necessitates a shift toward a truly personalized therapeutic strategy.

Our analysis uncovers that the future of precision psychiatry lies at the convergence of multiple scientific disciplines, where AI serves as the essential catalyst for integration. AI and ML possess the unique capability to synthesize complex, multi-omics data (like pharmacogenetics testing results on CYP450 polymorphisms and pharmacodynamic genes, and real-time drug concentration data from therapeutic drug monitoring) with clinical and real-world evidence. This synergy enables the construction of dynamic, patient-specific predictive models that can forecast optimal drug selection and dosing before the first prescription is filled, effectively bypassing the protracted and often frustrating «trial-and-error» process. This transition from single-marker analysis to polygenic risk scores, validated by functional TDM data, represents the most immediate and logical frontier for digital health in MDD management.

Achieving this level of precision requires a concerted interdisciplinary research effort, uniting the expertise of pharmacologists, geneticists, mathematicians, and data scientists. Researchers must focus on developing and validating comprehensive predictive models that not only account for genetic predisposition and pharmacokinetics but also integrate emerging factors. This collaborative approach, which is increasingly recognized as vital across all medical fields, is crucial for overcoming the limitations of current broad diagnostic categories. Furthermore, the successful translation of these digital tools into clinical practice demands a focus on practical application and education, ensuring that future specialists are equipped with the necessary interdisciplinary thinking to leverage technologies like AI effectively. Through this synergistic and technology-driven approach, supported by robust validation and educational frameworks, the field can finally deliver truly targeted interventions that promise rapid and sustained improvement for patients with MDD.

## Data Availability

No new data were created or analyzed in this study. Data sharing is not applicable to this article.
